# Effect of direct adrenaline infusion into isolated rat heart on the induction of ventricular tachyarrhythmias^☆^

**DOI:** 10.1016/j.mex.2025.103231

**Published:** 2025-02-19

**Authors:** Ahmet Davut Aksu, Jana Hložková, Gerardo Enrique Abarca Ríos, Mohammed Naeem Malek, Roman Panovský, Grażyna Groszek, Petr Mokrý, Tomáš Kepák, Peter Scheer, Milan Sepši

**Affiliations:** a Department of Pharmacology and Toxicology, Faculty of Pharmacy, Masaryk University, Brno, Czech Republic; b International Clinical Research Centre, St. Anne's University Hospital, Brno, Czech Republic; c Department of Pharmacology, Pharmacy Faculty, University of Costa Rica, San Pedro, Costa Rica; d Department of Internal Cardio-angiology, Faculty of Medicine, Masaryk University, Brno, Czech Republic; e Faculty of Chemistry, Rzeszow University of Technology, Rzeszow, Poland; f Department of Chemical Drugs, Faculty of Pharmacy, Masaryk University, Brno, Czech Republic; g Department of Paediatric Oncology, Faculty of Medicine, Masaryk University, Brno, Czech Republic; h Department of Internal Cardiology, Faculty of Medicine, Masaryk University, Brno, Czech Republic

**Keywords:** Reperfusion arrhythmias, Langendorff apparatus, Epinephrine, Regional ischemia, Reperfusion injury, Direct adrenaline protocol

## Abstract

• In this study, hearts from 72 male *Wistar albino* rats were divided into two main protocols: a 40 min ischemia group (protocol A, *n* = 53) and 10 min ischemia group (protocol B, *n* = 19). Protocol A subdivided into 2 groups as a control group (*n* = 10) and adrenaline group (*n* = 43). Protocol B is subdivided into 2 groups as control group (*n* = 10) and adrenaline group (*n* = 9). Both adrenaline groups received the same dose of adrenaline.
• In protocol A, ventricular tachyarrhythmia (VTA) incidence was 0 % in controls but rose to 72 % in the adrenaline group (*p* < 0.01). Heart rates for the control and adrenaline groups in stabilization and reperfusion were 254±45 bpm and 247 ± 66 bpm, versus 277 ± 41 bpm and 651 ± 286 bpm, respectively.
• In protocol B, VTA incidence reached 100 % in both groups during reperfusion, with heart rates of 393 ± 29 bpm and 892±227 bpm for controls and 350 ± 49 bpm and 949 ± 116 bpm for the adrenaline group.
• These findings suggest that direct adrenaline administration into the heart in last 5 mins of the ischemic period and the 5 mins of in the reperfusion time increases the incidence of reperfusion-induced ventricular arrhythmias up to 72 % in protocol A. Protocol B hearts showed reperfusion-induced ventricular arrhythmias with 100 % incidence in both groups.

## Graphical abstract

**Figure:**
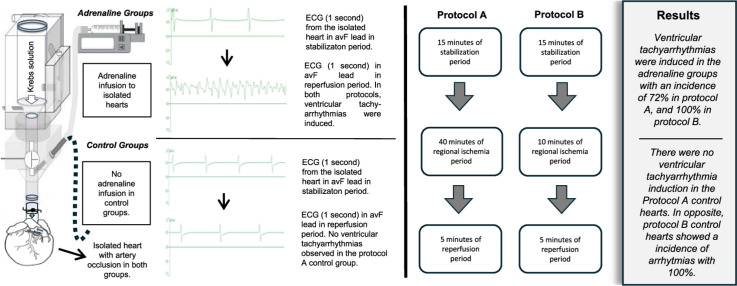

Specifications table

**Table:** 

Subject area:	Pharmacology, Toxicology and Pharmaceutical Science
More specific subject area:	Ex vivo heart research
Name of your protocol:	Direct adrenaline protocol
Reagents/tools:	Adrenaline 1mg/ml 1 ml ampule, (Zentiva, Czech Republic) Methylene Blue 319,9 g/mol 10 g (Roth Chemicals, Germany) Langendorff isolated perfused heart IH-5 core system (Harvard Apparatus, Massachusetts, USA). D-glucose; KCl; KH_2_PO_4_; MgSO_4_ x 7H_2_O; NaCl; NaHCO_3_; Na-pyruvate and CaCl_2_ (Merck Kga, Lach:Ner and Penta Chemicals). 95 % O_2_ and 5 % CO_2_ tube (Linde Gas). Benchtop pH meter (Orion Star A111, Thermo Scientific). pH electrode (Orion Triode Refillable Electrode, Thermo Scientific). Hugo Sachs Electronic Harvard Apparatus, March-Hugstetten, Germany. Starling Resistor for IH-5, (Harvard Apparatus). Plugsys module servo controller for perfusion (Hugo Sachs Elektronik, Harvard Apparatus March-Hugstetten, Germany). Medical Infusion pump (P9001, Onyx CZ) Isoheart v2.0 software (Hugo Sachs Elektronik GmbH). Circulating water bath (Optima TX150). Harvard Apparatus peristaltic pump p-70 (Massachusetts, USA) Tubings with different diameters (Harvard Apparatus, Massachusetts, USA) Aortic cannulas with different diameters (Harvard Apparatus, Massachusetts, USA)
Experimental design:	Isolated hearts are subjected to two different protocols, protocol A and protocol B. Protocol A consists of 15 mins of stabilization, 40 mins of regional ischemia, and 5 mins of reperfusion. Protocol B consists of 10 mins stabilization, 10 mins regional ischemia and 5 mins of reperfusion. During the last 5 mins of ischemia and 5 mins of reperfusion period, adrenaline infused directly to the hearts in the adrenaline group of both protocols. Control group hearts did not receive any substance in both protocols.
Trial registration:	Not applicable.
Ethics:	This study confirms that the experiment complied with the ARRIVE guidelines and was carried out in accordance with Guidelines for Animal Experimentation of Masaryk University with approval from the Institutional Animal Care and Use [approval number: MSMT-17,860/2022–4]. All procedures performed on animals were performed by guidelines of the Directive of the European Parliament and of the Council.
Value of the Protocol:	• The protocol simulates the clinical scenario of reperfusion injury. • Protocol increases the reperfusion-induced ventricular tachyarrhythmias up to 72 % after 40 min ischemia. • The protocol is suitable for testing both antiarrhythmic and proarrhythmic compounds.

## Background

Ventricular tachyarrhythmias (VTA) are the most serious arrhythmic complications of cardiovascular diseases. *ex vivo* rat heart models are widely used in the study of ischemia or reperfusion induced ventricular arrhythmias [[1], [2], [3]]. Developing experimental models to study ischemia-reperfusion (I-R) injury is vital for understanding the underlying mechanisms and enhancing the potential therapeutic strategies. Different *ex vivo* protocols are reported in literature where regional I-R injury is achieved. It is possible to induce a regional ischemia, mainly by occluding the left anterior descending (LAD) artery [3,4]. Regional or low flow type of ischemia model with 20 to 30 mins duration predominates in most studies, with the incidence of ventricular fibrillation (VF) ranging widely from 8 to 100 % during ischemia [5]. Initiation of VF in ischemia period may be promoted by lowering the K^+^ level in K-H solution (serum K^+^ concentration < 3.5 mmol/l), the addition of adrenaline into the K-H solution (administered continuously throughout the experiment), or combinations of low K^+^ levels, high Ca^2+^ concentrations (2.4 mmol/l), with the addition of noradrenaline (313 nmol/l) and adrenaline (75 nmol/l) [[6], [7], [8], [9]]. Most of these protocols induce VTA during the ischemia period. One study with the 10 mins of regional ischemia reported that the incidence of reperfusion induced VF was 90 % in unpaced and right atrium intact hearts [10]. In our experiment, we wanted to simulate the clinical scenario of the I-R injury, where the differentiations in adrenaline concentration play a major role during the reperfusion turnover. For this purpose, adrenaline was infused directly to the isolated heart in the specified time with gradual increase of the dose during ischemia-reperfusion turnover. To prove this, we examined the incidence of VTAs and the heart rate during stabilization, ischemia and reperfusion period in two different protocols. QRS and QT intervals were collected for analysis. The goal of this study is to describe an applicable model of reperfusion-induced VTA on isolated hearts for testing antiarrhythmic compounds and detection of pro-arrhythmic drugs in basic and preclinical research.

## Description of protocol

### Animals

Adult male *Wistar albino* rats weighing 300–600 g in age over six months were randomly selected and used for the experiment. An animal keeper randomized rats by drawing lots. Animals were housed in IVC boxes with continuous access to water and food, which consisted of rat feed pellets at the animal facility at the Faculty of Pharmacy, Masaryk University. All animal care and experimental procedures were performed according to the Guidelines for Animal Experimentation of Masaryk University with approval from the Institutional Animal Care and Use [approval number: MSMT-17,860/2022–4]. All procedures performed on animals were performed by guidelines of the Directive of the European Parliament and of the Council.

### Experiment groups and description of protocols

**Fig. 1:**
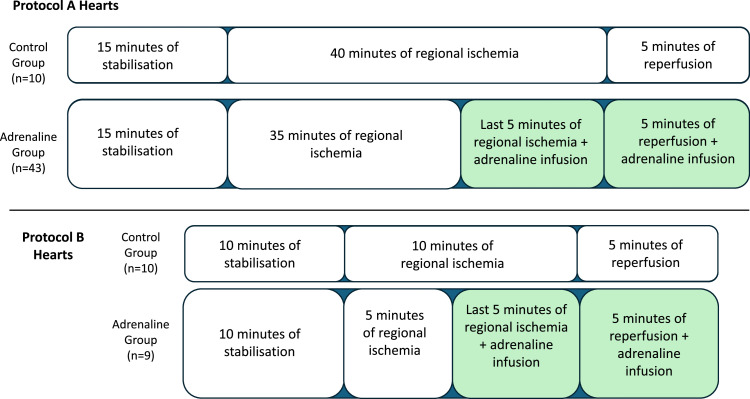
Schematic illustration of the experimental protocols and their division of groups. Protocol A hearts subjected to 15 mins of stabilization period, 40 mins of regional ischemia period and 5 mins of reperfusion period. Protocol B hearts subjected to 10 mins of stabilization period, 10 mins of ischemia period and 5 mins of reperfusion period. The subgroups of Protocol A and B, control and adrenaline, are given in the table with the number of hearts. Green boxes indicate the time and duration of the adrenaline infusion in adrenaline groups of protocol A and protocol B.

Isolated hearts removed from the 72 anaesthetized male *Wistar albino* rats with thoracotomy and divided into 2 main protocols as 40 min regional ischemia group (protocol A) (*n* = 53), and 10 min regional ischemia group (protocol B) (*n* = 19). Protocol A subdivided into 2 groups as a control group (*n* = 10) and adrenaline group (*n* = 43). Protocol B subdivided into 2 groups as a control group (=10) and adrenaline group (*n* = 9). Adrenaline group hearts received the infusion of adrenaline during the last 5 min of ischemia and the 5 mins of reperfusion period. Control group hearts did not receive adrenaline and were subjected to regional ischemia with same duration of stabilization, ischemia and reperfusion time depending on the protocol (Fig. 1).

### Isolation and perfusion of the hearts

**Fig. 2:**
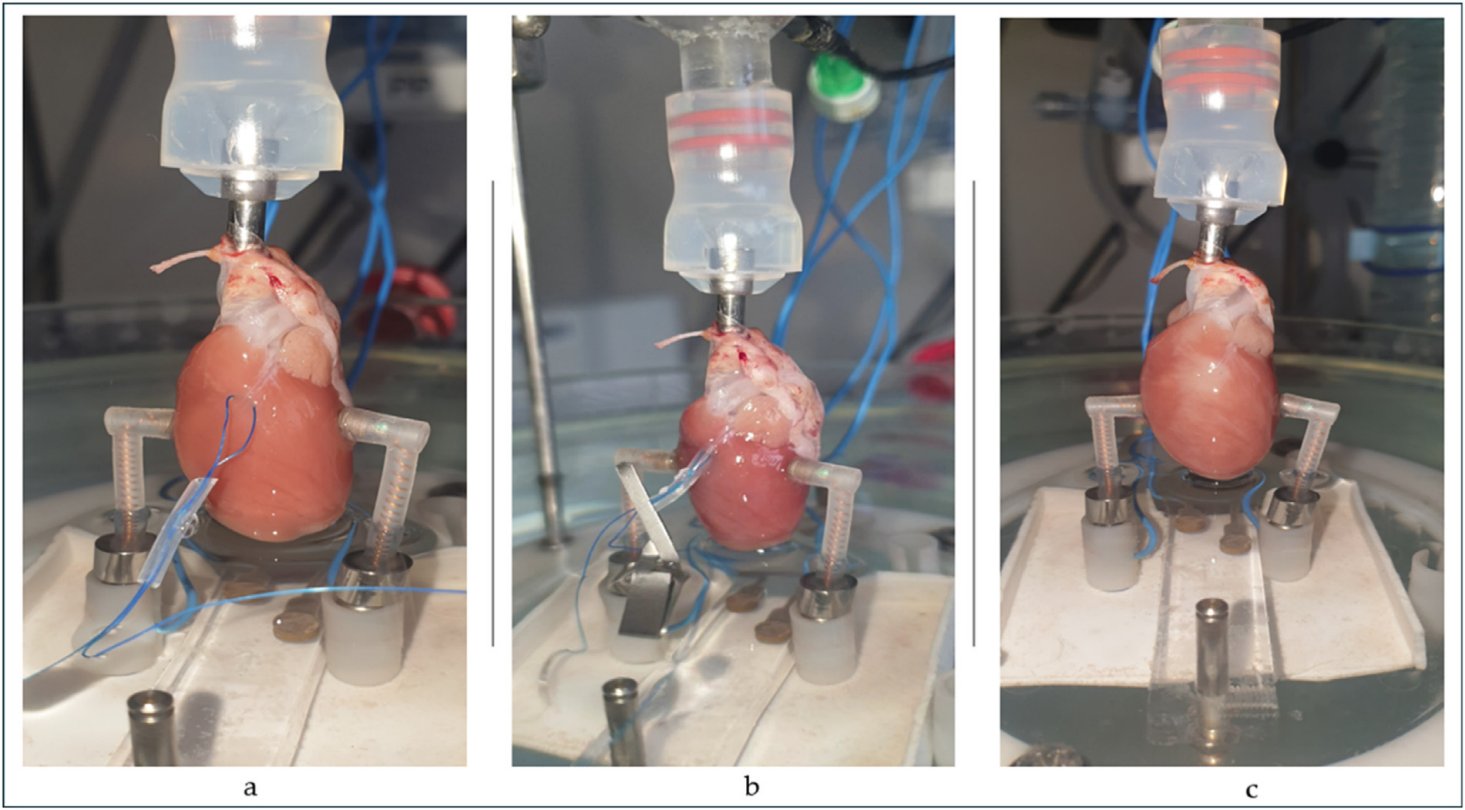
Preparation of left anterior descending artery occlusion (1a), application of occlusion (1b) and removal of occlusion (1c) on isolated beating rat heart in the Langendorff apparatus.

Rats were anaesthetised under a Vetequip inhalation anesthetic system and vaporizer (Livermore, CA, USA) with 2.5 % isoflurane. Anaesthesia continued until attenuation of loss of retinal reflexes and pedal reflexes (toe pinch). Later, 0.5 ml of heparin (5000 IU/ml, Zentiva, Czechia) was administered intraperitoneally. Later the rats were decapitated. Hearts excised from the body, placed into ice-cold saline immediately. The cannula inserted to the aorta and positioned between the brachiocephalic artery and the left common carotid artery. After cannulation, *truncus pulmonalis* is pierced to provide an outflow from the right ventricle. Langendorff isolated perfused heart IH-5 core system (Harvard Apparatus, Massachusetts, USA) used in this study for the perfusion of the hearts. Krebs solution used as a perfusate in the composition of (mmol/L) d-glucose 5.55; KCl 4.70; KH_2_PO_4_ 1.18; MgSO_4_ x 7H_2_O 1.1765; NaCl 118.00; NaHCO_3_ 24.88; Na-pyruvate 2.00 and CaCl_2_ 2.52. The chemicals were obtained from Merck Kga, Lach:Ner and Penta Chemicals. Before the experiment, the solution was saturated with 95 % O_2_ and 5 % CO_2_ (Linde Gas). A benchtop pH meter (Orion Star A111, Thermo Scientific) and pH electrode (Orion Triode Refillable Electrode, Thermo Scientific) were used to measure pH. Calibration of pH done with three different calibration solutions (pH 4.006 buffer, pH 6.865 buffer, pH 9.180 buffer, Thermo Scientific). After calibration of the electrode, pH was continuously monitored to keep it at 7.4 and maintained by adding 1 mol/l HCl or 1 mol/l NaOH (Merck) into the solution. Temperature of the system set to 37.6 °C using a circulating bath at (Optima TX150). Constant perfusion pressure set to 80 mmHg (Perfusion Pressure Control with Starling Resistor for IH-5, Harvard Apparatus). Backflow from the aortic block to the reservoir was provided during all experiment protocols in all hearts by using the servo controller (Hugo Sachs Elektronik, Harvard Apparatus) in direct mode. LAD artery was ligated with a monofil suture (USP 3–0) and a silicone tubing occluded on the artery using a micro-bulldog clamp. Ischemic zone confirmed by the injection of methylene blue (MB) (Roth Chemicals, Germany), after the occlusion. MB dissolved in saline and injected to the aortic block of Langendorff apparatus. After injection, MB dispersed in Krebs solution, perfusated to the isolated heart and washed out. The Ischemic zone was approved by the observation of pink- pale tissue, while the non-ischemic zone tissue was in dark blue colour. Ischemia was followed by 5 mins of reperfusion by simply removing the silicon tubing (Fig. 2)

### Adrenaline dosage and administration

**Fig. 3:**
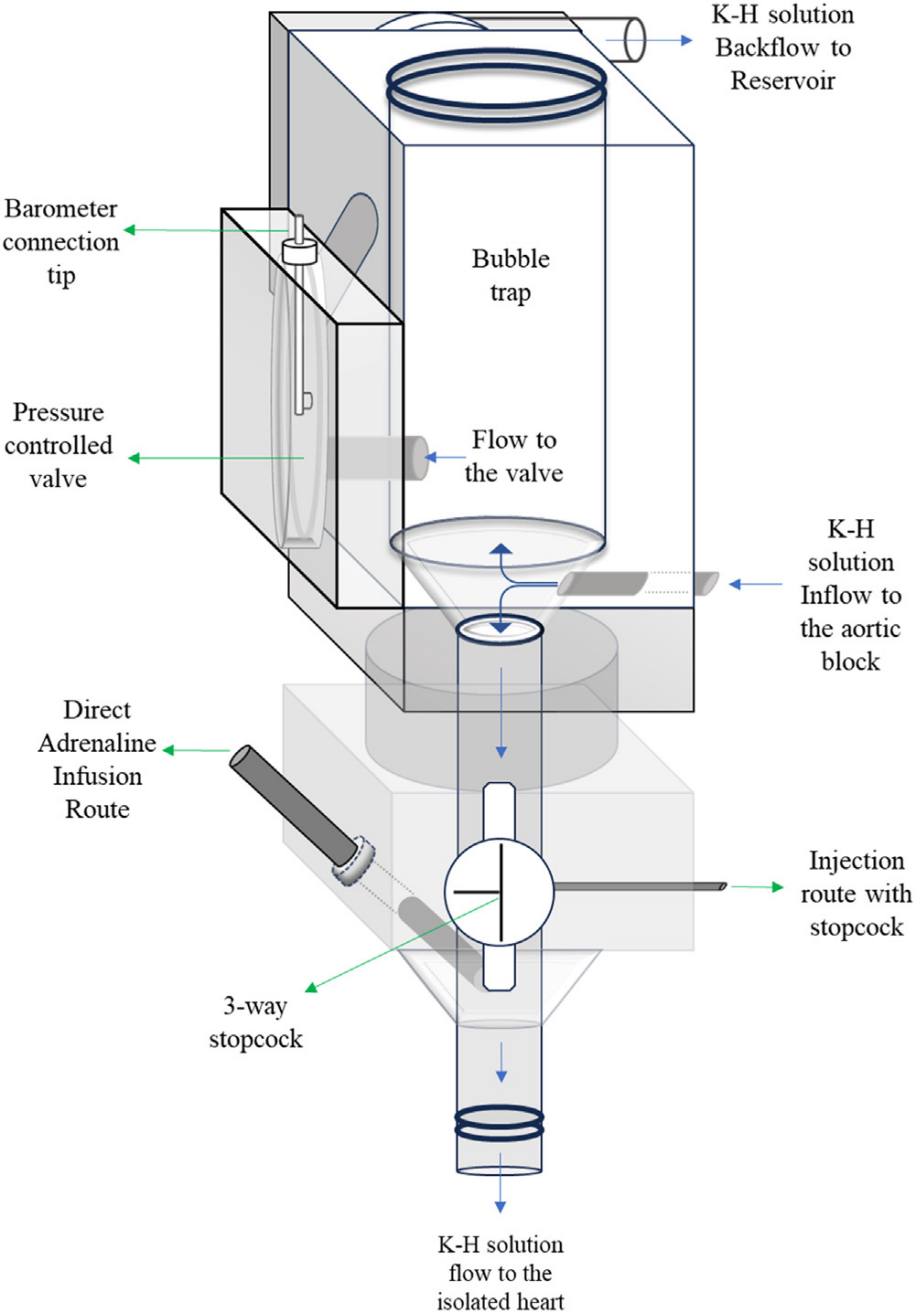
Illustration of the aortic block of the Langendorff apparatus (Harvard Apparatus, Massachusetts, USA). Constant perfusion pressure is provided to the heart by setting the connected barometer (see barometer connection tip) to 80 mmHg and observing the continuous backflow from the aortic block to the reservoir. With this, the pressure controlled half-membrane valve supplies the pressure from the barometer to the aortic block. Direct infusion route is independent from the stopcock hence provides an administration of adrenaline at specified time and dose to the heart.

In the adrenaline groups of both protocols, adrenaline was administered for 10 mins using an injection pump (P9001, Onyx CZ) during the last 5 mins of the ischemia period and the 5 mins of reperfusion time. In the last 5 mins of the ischemia period, adrenaline (Adrenalin 1 mg/ml inj. ampule, Zentiva) concentration was 0.025 mg/ml, and the infusion rate was 2.5 ml/h (0.042 ml/min). During the reperfusion period, adrenaline infusion rate was increased to 5.0 ml/h (0.084 ml/min). In our Langendorff system, the direct application route in the aortic block was used to infuse adrenaline to the isolated heart (Fig. 3).

### ECG recording and analysis

**Fig. 4:**
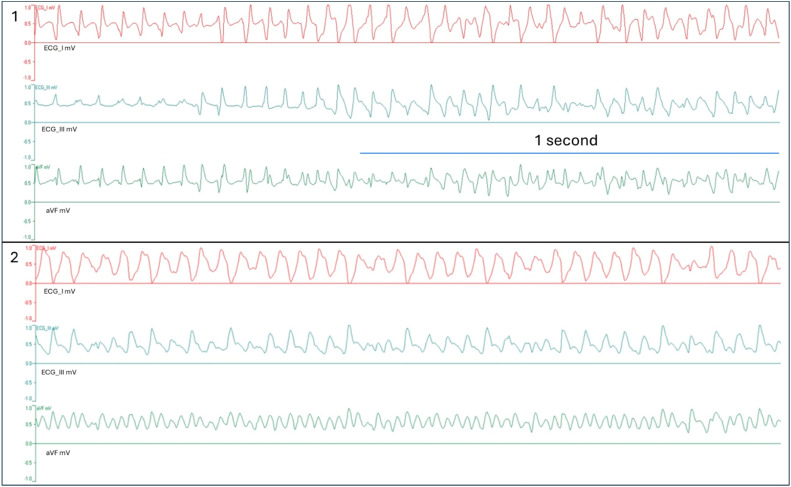
ECG complexes from ECG I, ECG III, and aVF leads in millivolt (mV) from 2 isolated hearts (heart 1 and heart 2) in the protocol A adrenaline group during the reperfusion period. Electrocardiograms were captured in 2.048-second intervals.

Surface electrocardiogram (ECG) recordings were listed using standard four “limb” electrodes (Input Boxes for Multi-ECG and Multimap Measurements on IH-5, Harvard Apparatus) throughout the experiment. “Hand” electrodes were placed on the left and right ventricular walls, and the “hindlimb” electrodes were conductively connected under the heart apex with K-H solution. Electrodes were always positioned in the same location to prevent artefacts. Recording of ECG started with hanging the heart into the system. ECG lines were obtained using the software Isoheart v2.0 (Hugo Sachs Elektronik, Germany) and ECG recordings analysed in all periods in both groups. ECG strips obtained from I, III, aVF and aVR leads. The formation of the ECG complexes in lead I, lead III, and aVF lead from two isolated hearts are given in Fig. 4.

QRS wave and QT wave intervals were measured using ImageJ software (Wisconsin, USA) [11]. ECG strips were captured in a 2.048-second window in Isoheart software during the stabilization period and reperfusion period. Given the challenge in measuring the intervals of QRS and QT during VTAs, analyses were made when VTA was not present throughout a period of 5 mins of reperfusion [12]. Scales in ImageJ are arranged according to the distance in pixels: 1868, known distance: 2.048 ms, and pixel aspect ratio: 1.0. Unit of length is set to milliseconds (ms), and the global scale was selected. A value of 0.9121 pixels/ms applied to all ECG strips in the measurements. The QRS interval is calculated by highlighting the distance between the beginning of Q wave and the end of S wave in both periods. The same method applied to measure the interval of QT complex by highlighting the distance between the beginning of Q wave and the end of T wave in both periods.

### Evaluation of heart rate and ventricular tachyarrhythmias

**Fig. 5:**
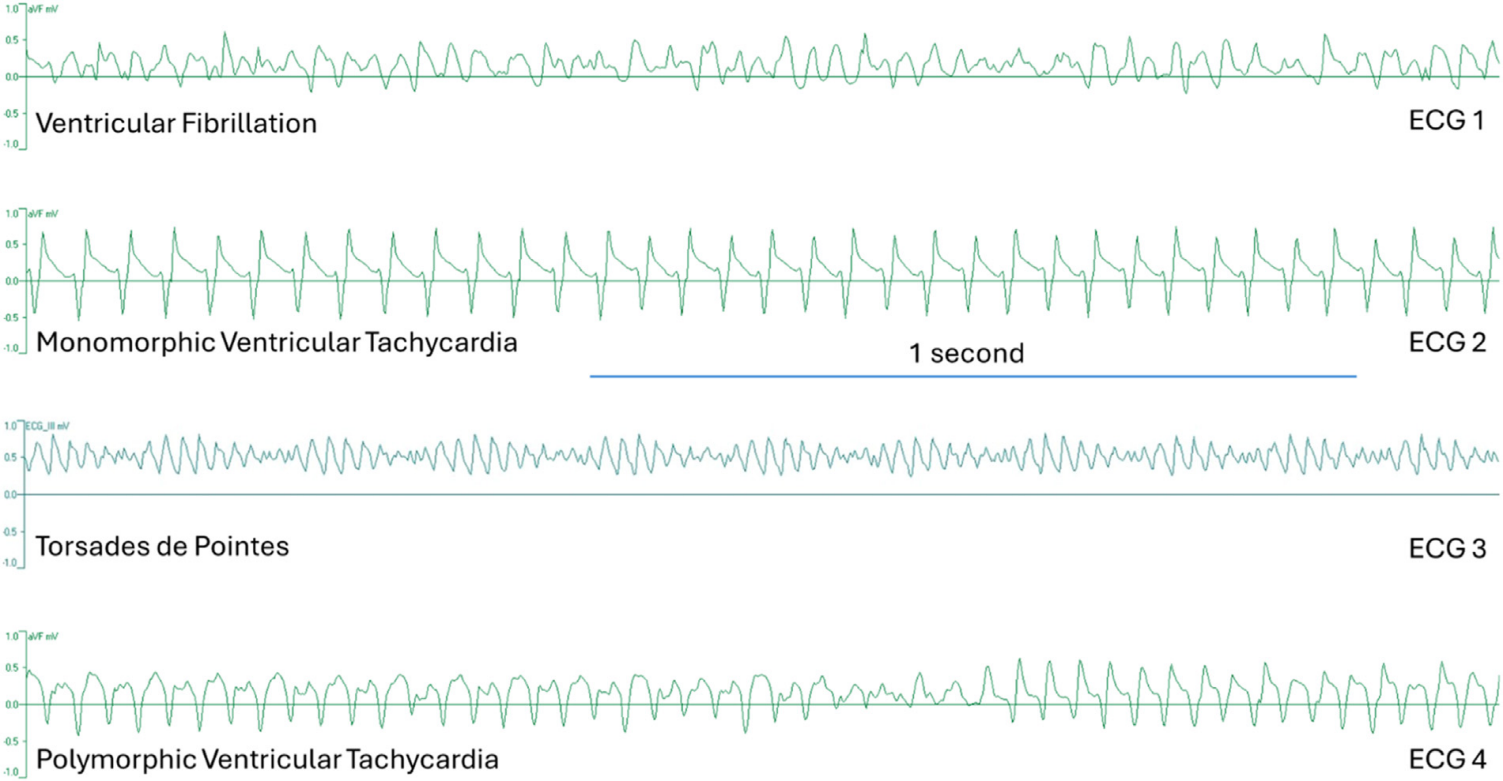
ECG strips from aVF (mV) and ECG_III leads in 4 different hearts with the indicated diagnosis. ECG 1 shows the readout diagnosed as ventricular fibrillation (VF). ECG 2 shows the readout diagnosed as monomorphic ventricular tachycardia. ECG 3 shows the readout identified as Torsades de Pointes (TdP). ECG 4 shows the readout identified as polymorphic ventricular tachycardia (PVT). ECG 1, ECG 2 and ECG 4 were obtained from protocol A hearts and ECG 3 was obtained from protocol B heart. All the ECG strips were captured in the reperfusion period. Identification of VF, PVT and TdP during the data analysis was done in accordance with the Lambeth conventions (II).

A ventricular complex in the ECG was defined as a single discrete complete electrical event in the ventricle. Q and T waves were not usually identifiable in ECG strips, as it mentioned in the Lambeth conventions (II) [12]. Heartbeat per minute (bpm) calculated by counting the R-R intervals per minute. In every heart, heart rate was calculated in the last minute of stabilization period, during the last 5 mins of ischemia and 5 mins of reperfusion. Mean heart rate with standard deviation (SD) was calculated from all values in the stabilization, last 5 mins of ischemia and 5 mins of reperfusion time depending on the groups. Duration of VTAs is classified as VTAs longer than 11 ss or shorter than 11 ss. When the VTA durations were <11 ss, then the heartbeat/min was not entered as arbitrary 1000 bpm and calculated manually. When the VTA durations were >11 ss, the heart rate was arbitrarily determined as 1000 bmp due to its continuous occurrence and the impossibility of differentiating the consecutive QRS complexes during the VTA. Oscillations formed in smaller voltage with the start of negative deflection and end with positive deflections but did not interfere with the VTA identification. In our study, occurrence of ventricular premature beats, ventricular bigeminy, salvos, and monomorphic ventricular tachycardia weren't classified as ventricular tachyarrhythmia. Therefore, their occurrence was out of interest. Torsades de pointes (TdP), polymorphic ventricular tachycardia (PVT) and ventricular fibrillations were considered as VTA, and their occurrence was evaluated during the reperfusion period. PVT, TdP and VF were identified based on Lambeth conventions (II) as shown. (Fig. 5) [12]. PVT is identified by at least four successive ventricular complexes where the interval between peaks, the height, or the shape varies, with at least one of these factors changing progressively. TdP is identified as a specific kind of PVT with the ventricular complex appearing to twist around the isoelectric baseline, where at least four consecutive ventricular complexes progressively change in height, though the interval between peaks may either stay the same or change. VF is identified by at least four successive ventricular complexes with no pauses in between, where the shape, interval between peaks, and height all change, but unlike PVT, these changes do not progress in a specific pattern. It was reported that defining the VF was challenging, and it can be difficult to identify a ventricular tachyarrhythmia in experiments, especially when the arrhythmia self-terminates swiftly after onset [12]. Parallel with this, we classified PVT and VF as ventricular tachyarrhythmias (VTA) together with TdP. The identifiable T waves were not present in PVT and VF. Lastly, monomorphic ventricular tachycardias weren't considered as VTAs due to their unvarying height and/or intrinsic shape with rapid, short, repetitive and uniform peak-peak intervals.

### Coronary flow measurements

Coronary flow (CF) was measured on 20 hearts in adrenaline group of protocol A and 6 hearts in adrenaline group of protocol B. CF measured by placing a small beaker under the hanging heart in stabilization and at the end of reperfusion period using a 25:0.5 ml graduated cylinder for one minute.

### Exclusion criteria

Hearts with ventricular tachyarrhythmias in stabilization and ischemia period were removed from the study. Hearts with <200 beats/min during stabilization were excluded as reported before [14]. Hearts falling out of the coronary flow range between 8 and 20 ml/min in stabilization were excluded. Hearts due to wrong occlusion and technical issues such as pH malfunction, multiple tries of the cannulation, unreadable ECG and overwritten data were also removed from the study.

### Statistical analysis

Data collected from the study were analysed using the IBM SPSS statistics v26. Experimental data was entered into the software, and descriptive statistics, independent samples *t*-test (Student's *t*-test), and boxplot figures were obtained. All data are in mean ± standard deviation (SD) format unless otherwise noted.

## Protocol validation

### Results

**Fig. 6:**
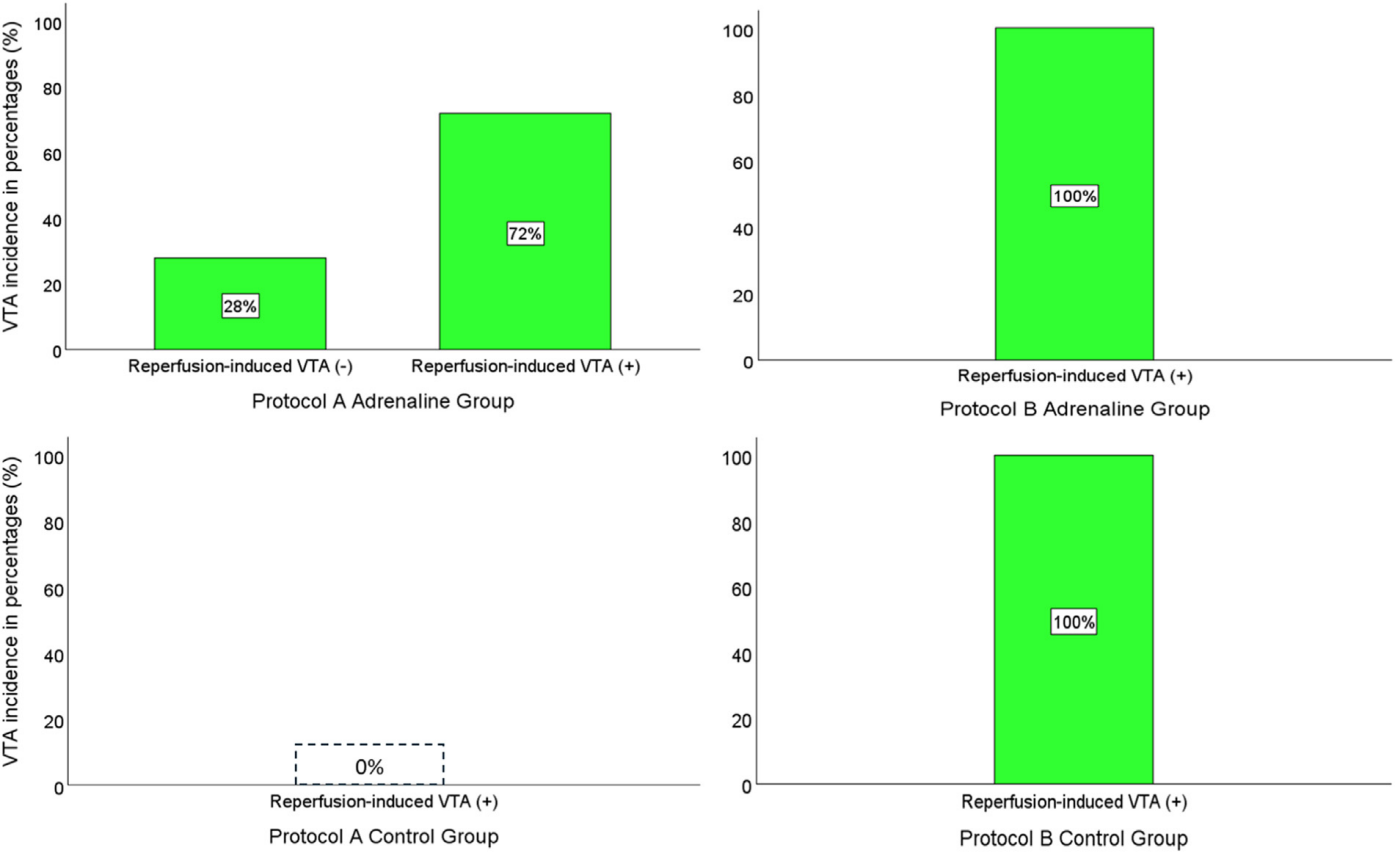
Data in bar charts showing the incidence of ventricular tachyarrhythmias (VTA) of adrenaline and control groups in protocol A and protocol B hearts. “(+)” indicates the occurrence of the VTA in the bar chart. “(-)” stands for the no occurrence of VTA.

Hearts in protocol A adrenaline group showed a 72 % incidence of ventricular tachyarrhythmias (VTA) while the control group showed a 0 % of VTAs (*p* < 0.001). A total number of 43 hearts were included in the adrenaline group of protocol A. 12 hearts in the adrenaline group of protocol A(28 %) did not show any VTAs while the remaining 31 hearts (72 %) had VTAs. In protocol B hearts, the incidence of the VTAs were 100 % in both groups (Fig. 6)

**Fig. 7:**
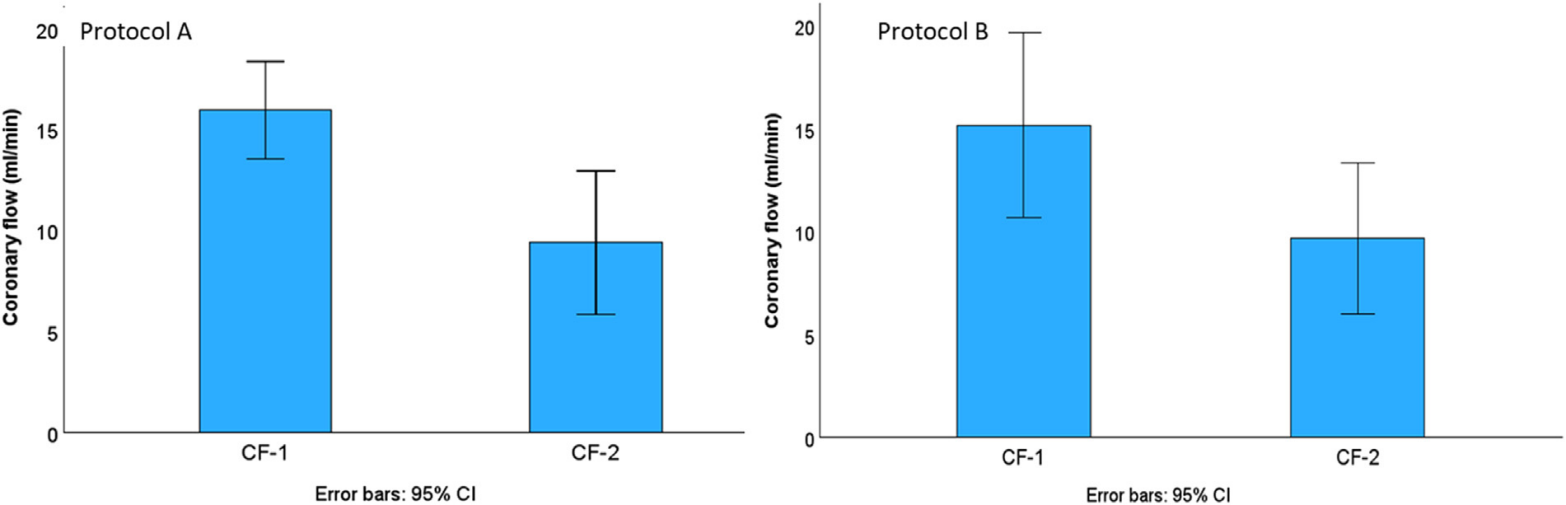
Coronary flow (ml/min) of the hearts in adrenaline group in protocol A and protocol B. Boxplot graphs show the coronary flow measurements in the stabilization period and in the end of the reperfusion period. CF-1: Coronary flow in stabilization. CF-2: Coronary flow at the end of reperfusion. CI: Confidence index.

In protocol A adrenaline group, the value (mean ± SD) of coronary flow rate (ml/min) isolated hearts in stabilization was 16 ± 5 ml/min, and 9 ± 7 ml/min in reperfusion period. In protocol B, the coronary flow rate in isolated hearts in stabilization was 15 ± 4 ml/min, and 9 ± 3 ml/min in reperfusion (Fig. 7).

**Fig. 8:**
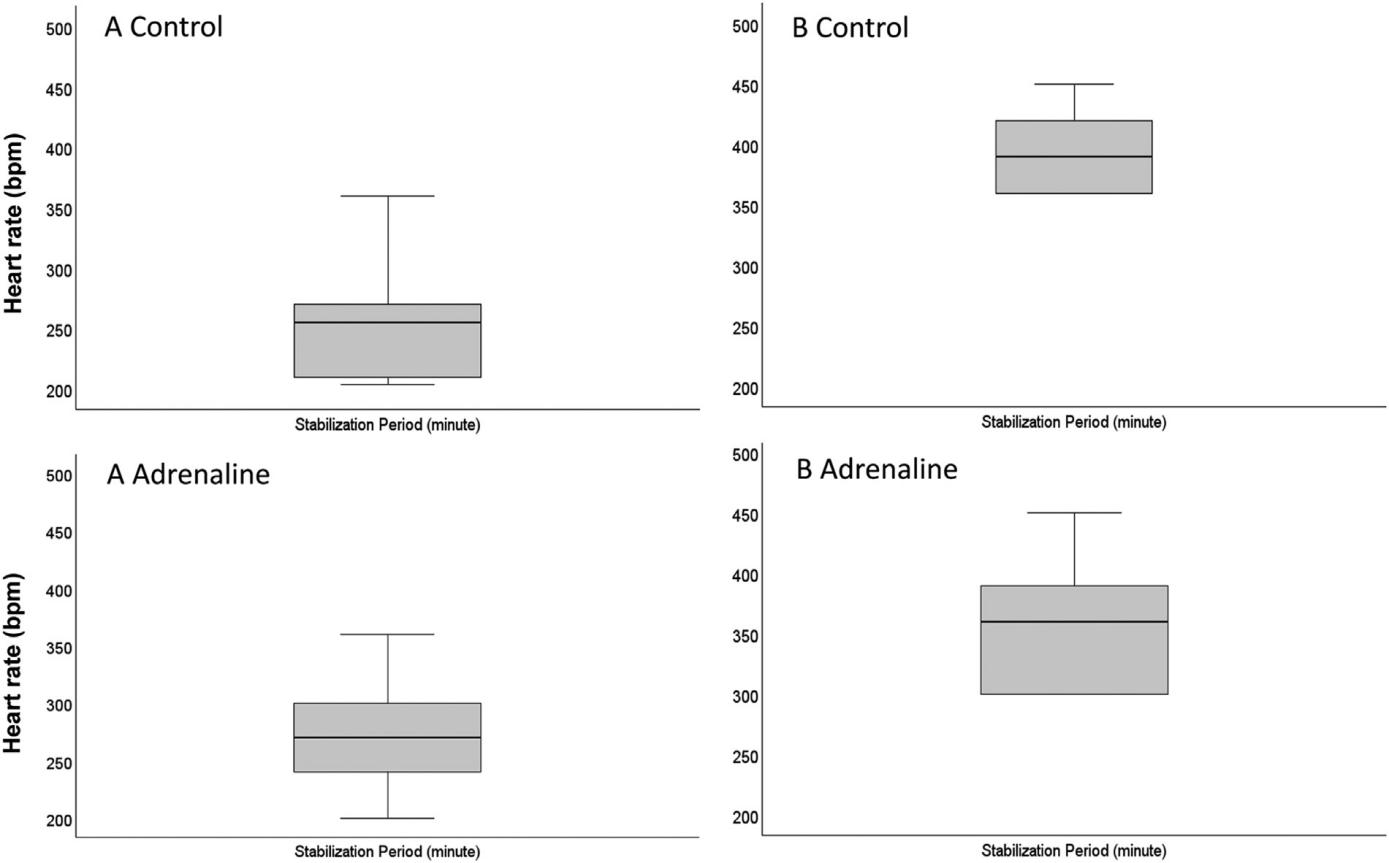
Boxplots of heart rate (BPM) in adrenaline and control groups in protocol A and protocol B hearts during the stabilization period. Bpm: beat per minute.

**Fig. 9:**
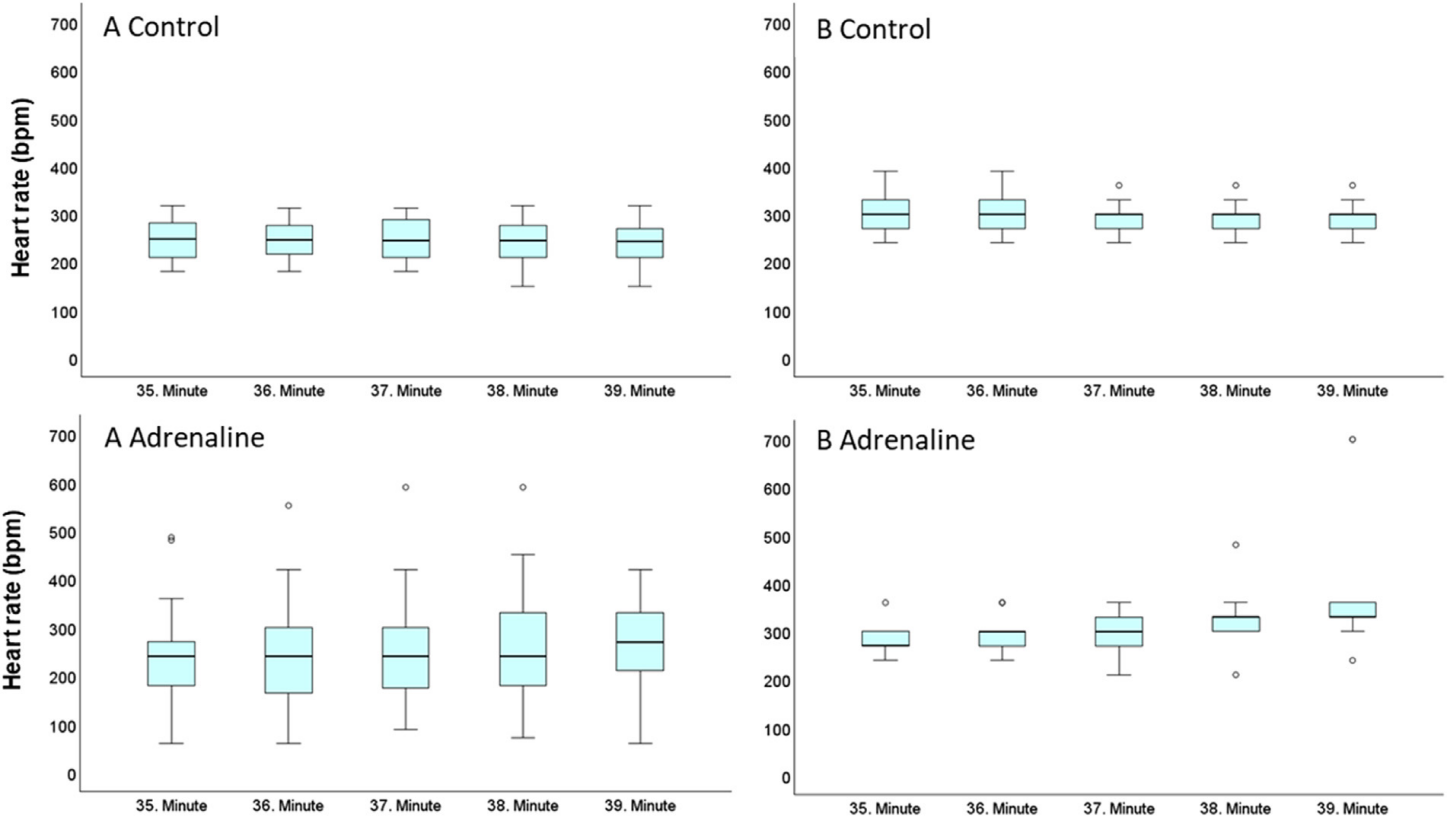
Boxplots of heart rate (BPM) in adrenaline and control groups in protocol A and protocol B hearts during the last 5 min of ischemia period. Symbol “°” indicates the single-deviated heart rate value. Bpm: beat per minute.

**Fig. 10:**
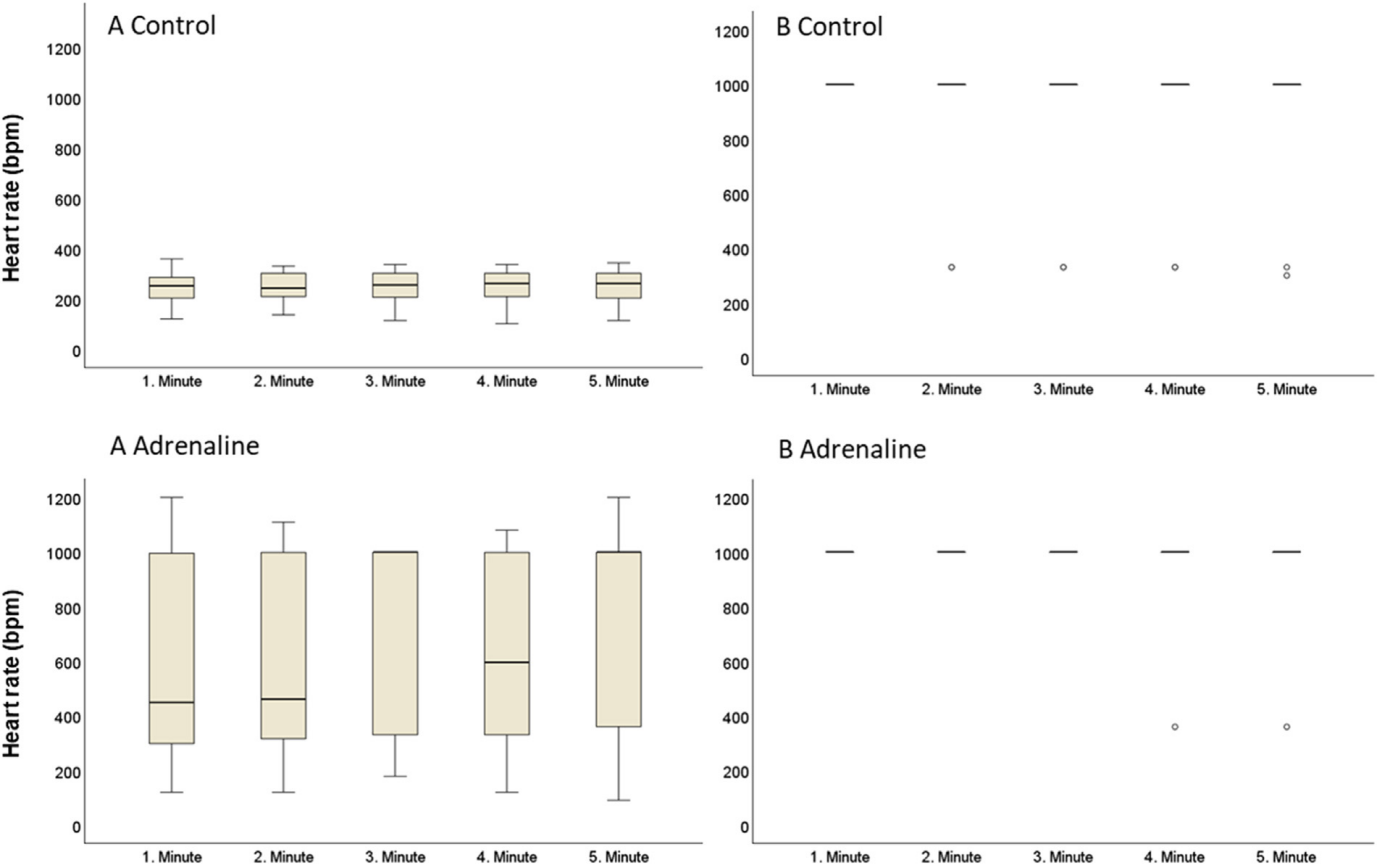
Boxplots of heart rate (BPM) in adrenaline and control groups in protocol A and protocol B hearts during the reperfusion period. Almost all of the protocol B hearts had a reperfusion-induced VTA. Symbol “°” indicates the single-deviated heart rate value of the hearts. Bpm: beat per minute.

Experiment results showed that the heart rate (mean ± SD) of the hearts in protocol A control group was 254 ± 45 bpm during the stabilization, 243 ± 47 bpm during the last 5 mins of ischemia, and 247 ± 66 bpm during the reperfusion period (Fig. 8, Fig. 9). Heart rates in the protocol A adrenaline group were 277 ± 41 bpm during stabilization, 245 ± 92 bpm during the last 5 mins of ischemia, and 651 ± 286 bpm during the reperfusion period. Difference between the heart rate in the groups of protocol A during the reperfusion period was statistically significant (*p* < 0.005) (Fig. 10). In protocol B, heart rate in control group during the stabilization was 393 ± 29 bpm, 298 ± 38 bpm during the last 5 mins of ischemia, and 892 ± 227 bpm in the reperfusion period. In the same protocol, hearts in adrenaline group had a 350 ± 49 bpm heartrate in stabilization, 319 ± 56 bpm during the last 5 mins of ischemia, and 949 ± 116 bpm in reperfusion period. In contrast to protocol A, the difference between the heart rate in the groups of protocol B was not statistically significant (Fig. 8, Fig. 9, Fig. 10).

**Fig. 11:**
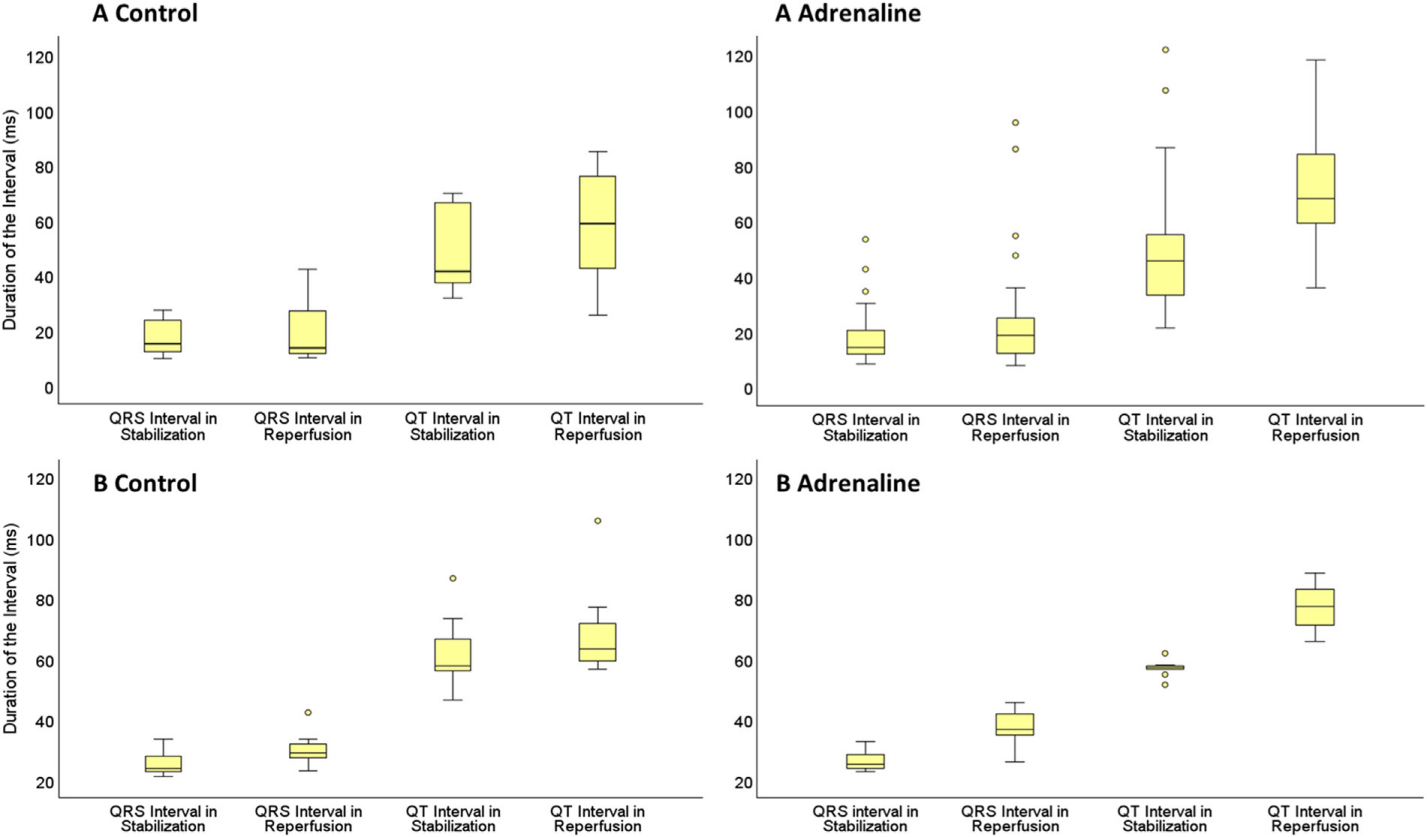
QRS wave and QT wave intervals (ms) of the of hearts in adrenaline and control groups in protocol A and protocol B hearts during the stabilization and reperfusion period. “A Control” stands for the hearts in the protocol A control group. “A adrenaline” stands for the hearts in the protocol A adrenaline group. “B Control” stands for the hearts in the protocol B control group. “B adrenaline” stands for the hearts in the protocol B adrenaline group.

In protocol A control group, the difference of the QT interval between the stabilization and reperfusion period was statistically significant which was same in the adrenaline group (*p* < 0.05) (Fig. 11). There were no significant differences in QRS intervals in both groups. In protocol B, the differences of the QRS wave and QT wave intervals between stabilization and reperfusion period were statistically significant (*p* < 0.05) (Fig. 11). By occurrence of 5-minute VTA, it was impossible to measure the QRS interval and QT interval of 2 hearts in protocol A and 2 hearts in protocol B. Due to this, 68 hearts were suitable for measuring the QRS and QT interval in total.

### Discussion

In this study, we proposed a new *ex vivo* model with direct adrenaline infusion to the heart in the specific period of ischemia and reperfusion for the first time in literature (Fig. 3). It is reported in the literature that the inclusion of 10 μM of adrenaline in Krebs medium induces 100 % VF, but this occurs during the ischemia [8]. It's known that the catecholamine levels increase in the heart during the ischemia-reperfusion (I-R) injury [13]. In pathological scenarios, the body naturally releases much higher levels of adrenaline. We wanted to mimic these intense physiological conditions by infusing adrenaline during a specific time window coinciding with the transition point between regional ischemia and reperfusion. in our protocols. The small size of the rat heart can limit the number of re-entry circuits that can exist simultaneously [14]. The high proportion of refractory tissue compared to the size of the ischemic zone increases the chance that re-entry circuits will encounter refractory tissue and terminate [15]. Consequently, ventricular fibrillation in the rat heart during the reperfusion is likely to self-terminate because the re-entry circuits can be interrupted by hitting refractory tissue, which was observed in our study. This also explains why ventricular arrhythmias might not be sustained as long as in the larger hearts [16]. Triggered activity induces increase in oxidative stress which plays a major role in the initiation of arrhythmias by I-R damage [17]. I-R injury can cause abnormal depolarizations in cardiac muscle cells due to changes in ion concentrations and channel functions [18]. Reperfusion reactivates calcium channels, causing calcium overload, and triggers potassium efflux, altering membrane potential [19,20]. These effects contribute to early afterdepolarizations (EADs) and delayed afterdepolarizations (DADs) [19].

In this study 152 hearts were used, and 77 hearts were selected for the analysis. The rest 75 hearts were removed depending on the exclusion criterias. Results obtained by performing 4 different ischemia-reperfusion injury protocols. Adrenaline received hearts in protocol A showed a significant increase in heart rate and VTA incidence during reperfusion compared to control group. Occurrence and durations of VTAs varied in the 5 mins of reperfusion time but mostly happened in the first minute with a duration longer than 11 ss. Besides, QRS wave and QT wave length were significantly increased in the adrenaline group during the reperfusion time. The reason can be that adrenaline paradoxically worsens the I-R injury and leads to higher tissue damage. Increased amount of calcium inside the cells keeps the membranes depolarised, hence contributing to slower and less synchronised ventricular depolarisation, manifesting as a prolonged QRS and QT interval. Apart from that, the reason why there were no VTA occurrences in the control group of the protocol A, can be the lack of adrenaline together with potassium protection during the 40 mins of ischemia period. It was reported that in normal Krebs solution, potassium concentration is much higher than normal, which can protect the heart from I-R injury [21]. It also reported a model with 100 % VF incidence in case of 2 mM potassium concentration in Krebs with the duration of 30 mins of regional ischemia [22]. On this basis, in protocol A, 5.88 mM of K was enough to prevent the VTAs in control group but not enough to prevent the VTAs in adrenaline group. In the control group of the protocol B, the incidence of the VTAs was 100 %, which was higher than reported [10]. In the adrenaline received hearts the incidence was also 100 %. The reason for the different VTA incidences in both protocols is because they differ in the duration of ischemia. Hearts under 40 mins ischemia are more vulnerable to necrosis and higher tissue damage than the 10 mins ischemia period. Results from the coronary flow measurements showed a decrease in the coronary flow after the 5 mins of reperfusion period compared to coronary flow values in stabilization in both protocols. The decrease in the coronary flow during the reperfusion can be also due to the loss of pulsatile flow and no myocardial contraction, which increases coronary resistance.

Another important finding from our study is the role of the heart rate difference between ischemia and reperfusion. We found that if the heart rate difference is <150 beats, there was no reperfusion induced VTAs in both protocols. Contrarily, when the heart rate difference is >150 beats, there was always reperfusion induced VTAs in both protocols. In our experiment methylene blue was used as in the previous studies as a dying agent to control the occlusion and visualize the size of the ischemic zone [23]. In the adrenaline group of the protocol A, 24 hearts didn't receive methylene blue and 10 of them did not show reperfusion-induced VTA. 19 hearts received methylene blue and 17 of them showed a reperfusion induced VTAs. For testing the effect of methylene blue on the 10 mins ischemia protocol, we applied methylene blue to the 5 hearts and 2 of them did not show reperfusion induced VTA. Our unpublished data showed that methylene blue applied to the 10 mins ischemia model hearts showed an antiarrhythmic effect while it was showing a proarrhythmic effect on 40 mins ischemia protocol. Lastly, we customized procedures for testing VTA suppressants or pro-arrhythmogenic agents and controlled the difference in the number of subjects between groups. The proportion effect size calculation allowed us to determine the smallest number of individuals needed to obtain statistically significant results for VTA suppression compared to the adrenaline group while sparing the use of live animals [24]. For instance, during the analysis of potential antiarrhythmic compounds with the 10 subjects in group (*n* = 10) group, it's possible to have 2 subjects with VTA occurrence and 8 subjects with suppressed VTA.

In this study, we introduced two new protocols for reperfusion-induced ventricular tachyarrhythmias in isolated hearts. Protocols A and B describe a two-step direct (to coronary ostia) administration of adrenaline to the coronary ostia, leading to VTA incidences of up to 72 % and 100 %, respectively.

## Limitations

Generally, an isolated heart is denervated. When there is a fast ventricular monomorphic tachycardia, bradycardia or VTA, the systemic perfusion pressure, preload, and afterload are stable, which is a contra-situation in real patients. Plus, the aortic pressure was maintained constantly at 80 mmHg, separated from the ventricular contractions during VTA. This is in contrast with the clinical situation of VTA, where the aortic pressure drops drastically together with the coronary flow and cardiac output. Another limitation is the occlusion of the left anterior descending artery. Since this process is done by hands manually, the ischemic zone size might vary and that can lead to different incidences of VTA among the hearts.

## CRediT authorship contribution statement

**Ahmet Davut Aksu:** Conceptualization, Methodology, Software, Data curation, Writing – original draft. **Jana Hložková:** Conceptualization, Methodology, Supervision, Visualization, Writing – review & editing. **Gerardo Enrique Abarca Ríos:** Data curation, Software, Writing – review & editing. **Mohammed Naeem Malek:** Data curation, Writing – review & editing. **Roman Panovský:** Investigation. **Grażyna Groszek:** Investigation. **Petr Mokrý:** Visualization. **Tomáš Kepák:** Validation. **Peter Scheer:** Supervision, Methodology, Conceptualization, Software. **Milan Sepši:** Visualization, Validation.

## Declaration of competing interest

The authors declare that they have no known competing financial interests or personal relationships that could have appeared to influence the work reported in this paper.

## Data Availability

Data will be made available on request.

## Appendix Supplementary materials

Image, application 1

## References

[1] Vidavalur R., Swarnakar S., Thirunavukkarasu M., Samuel S.M., Maulik N. (2008). Ex vivo and In vivo approaches to study mechanisms of cardioprotection targeting ischemia/reperfusion (I/R) injury: useful techniques for cardiovascular drug discovery. Curr. Drug. Discov. Technol.

[2] Neckář J., Alánová P., Olejníčková V., Papoušek F., Hejnová L., Šilhavý J. (2021). Excess ischemic tachyarrhythmias trigger protection against myocardial infarction in hypertensive rats. Clin. Sci.

[3] Lindsey M.L., Bolli R., Canty J.M., Du X.J., Frangogiannis N.G., Frantz S., Gourdie R.G., Holmes J.W., Jones S.P., Kloner R.A., Lefer D.J., Liao R., Murphy E., Ping P., Przyklenk K., Recchia F.A., Schwartz Longacre L., Ripplinger C.M., Van Eyk J.E., Heusch G (2018). Guidelines for experimental models of myocardial ischemia and infarction. Am. J. Physiol. Heart Circ. Physiol..

[4] Al-Awar A., Almási N., Szabó R., Takacs I., Murlasits Z., Szűcs G. (2018). Novel potentials of the DPP-4 inhibitor sitagliptin against ischemia-reperfusion (I/R) injury in rat ex-vivo heart model. Int. J. Mol. Sci..

[5] Curtis M.J. (1998). Characterisation, utilisation and clinical relevance of isolated perfused heart models of ischaemia-induced ventricular fibrillation. Cardiovasc. Res..

[6] Marshall R.J., Muir A.W., Winslow E. (1981). Development of a severe model of early coronary artery ligation-induced dysrhythmias in the anaesthetized rat. Br. J. Pharmacol..

[7] Farkas A., Curtis M.J. (2002). Limited antifibrillatory effectiveness of clinically relevant concentrations of class I antiarrhythmics in isolated perfused rat hearts. J. Cardiovasc. Pharmacol..

[8] Manning A.S., Kinoshita K., Buschmans E., Coltart D.J., Hearse D.J. (1985). The genesis of arrhythmias during myocardial ischemia. Dissociation between changes in cyclic adenosine monophosphate and electrical instability in the rat. Circ. Res..

[9] Stables C.L., Curtis M.J. (2009). Development and characterization of a mouse in vitro model of ischaemia-induced ventricular fibrillation. Cardiovasc. Res. Volume.

[10] Bernier M., Curtis M.J., Hearse D.J. (1989). Ischemia-induced and reperfusion-induced arrhythmias: importance of heart rate. Am. J. Physiol..

[11] Schneider C.A., Rasband W.S., Eliceiri K.W. (2012). NIH image to ImageJ: 25 years of image analysis. Nat. Methods.

[12] Curtis M.J., Hancox J.C., Farkas A., Wainwright C.L., Stables C.L., Saint D.A. (2013). The Lambeth Conventions (II): guidelines for the study of animal and human ventricular and supraventricular arrhythmias. Pharmacol. Therap..

[13] Schömig A., Dart A.M., Dietz R., Mayer E., Kübler W. (1984). Release of endogenous catecholamines in the ischemic myocardium of the rat. Part A: locally mediated release. Circ. Res..

[14] Jalife J. (2000). Ventricular fibrillation: mechanisms of initiation and maintenance. Annu. Rev. Physiol..

[15] Waks J.W., Josephson M.E. (2014). Mechanisms of atrial fibrillation - reentry, rotors and reality. Arrhythm. Electrophysiol. Rev.

[16] Hundahl L.A., Tfelt-Hansen J., Jespersen T. (2017). Rat models of ventricular fibrillation following acute myocardial infarction. J. Cardiovasc. Pharmacol. Ther..

[17] Akar J.G., Akar F.G. (2007). Regulation of ion channels and arrhythmias in the ischemic heart. J. Electrocardiol..

[18] Hausenloy D.J., Yellon D.M. (2013). Myocardial ischemia-reperfusion injury: a neglected therapeutic target. J. Clin. Invest..

[19] Hearse D.J. (1991). Reperfusion-induced injury: a possible role for oxidant stress and its manipulation. Cardiovasc. Drugs Ther..

[20] Murphy E., Steenbergen C. (2008). Mechanisms underlying acute protection from cardiac ischemia-reperfusion injury. Physiol. Rev..

[21] Clements-Jewery, H. and Curtis, M.J. (2014). The Langendorff Preparation. In Manual of Research Techniques in Cardiovascular Medicine (eds H. Ardehali, R. Bolli and D.W. Losordo).

[22] Curtis M.J., Hearse D.J. (1989). Ischaemia-induced and reperfusion-induced arrhythmias differ in their sensitivity to potassium: implications for mechanisms of initiation and maintenance of ventricular fibrillation. J. Mol. Cell Cardiol..

[23] Dai W., Amoedo N.D., Perry J., Le Grand B., Boucard A., Carreno J. (2022). Effects of OP2113 on myocardial infarct size and No reflow in a rat myocardial ischemia/reperfusion model. Cardiovasc. Drugs Ther..

[24] Kohn JS Michael. Proportions – Effect size | sample size calculators [Internet]. [cited 2024 Feb 27]. Available from: https://sample-size.net/proportions-effect-size/.

